# Aktiv und zufrieden? Körperliche Aktivität und berufsbezogene Lebensqualität bei Psycholog:innen

**DOI:** 10.1007/s00278-023-00645-x

**Published:** 2023-02-07

**Authors:** Antonia Bendau, George Oliver Gerz, Andreas Ströhle, Moritz Bruno Petzold

**Affiliations:** 1 Charité – Universitätsmedizin Berlin, corporate member of Freie Universität Berlin and Humboldt Universität zu Berlin, Klinik für Psychiatrie und Psychotherapie, CCM, Charitéplatz 1, 10117 Berlin, Deutschland; 2 HMU Health and Medical University Potsdam, Potsdam, Deutschland; 3 Universität Potsdam, Potsdam, Deutschland

**Keywords:** Psychotherapeuten, Emotionaler Stress, Psychische Gesundheit, Sport, Training, Psychotherapists, Emotional stress, Mental health, Sports, Exercise

## Abstract

**Hintergrund:**

Klinisch tätige Psycholog:innen sind in ihrer Arbeit mit Menschen mit psychischen Erkrankungen oft zahlreichen Stressoren ausgesetzt; diese können sich in psychischer Belastung und einer verringerten Lebensqualität niederschlagen. Körperliche Aktivität könnte eine protektive Ressource darstellen, diesbezüglich fehlen jedoch bisher empirische Befunde.

**Methoden:**

Im Rahmen einer querschnittlichen Online-Befragung über SoSci-Survey (Januar bis April 2020) wurden die körperliche Aktivität und die berufsbezogene Lebensqualität (positive Dimension: Mitgefühlszufriedenheit; negative Dimension: Mitgefühlsmüdigkeit) einer Gelegenheitsstichprobe aus 443 klinisch tätigen Psycholog:innen mithilfe etablierter Selbstberichtfragebogen (International Physical Activity Questionnaire – Short Form [IPAQ-SF], Professional Quality of Life Scale [ProQOL]) erfasst.

**Ergebnisse:**

Der Großteil der befragten Psycholog:innen erfüllte das von der Weltgesundheitsorganisation empfohlene Mindestmaß an körperlicher Aktivität. Die Mitgefühlszufriedenheit war im Schnitt relativ hoch, während eher geringe bis moderate Ausprägungen an Burn-out-Symptomatik sowie sekundärem traumatischem Stress (Facetten der Mitgefühlsmüdigkeit) berichtet wurden. Außer vereinzelten kleinen negativen Korrelationen zwischen körperlicher Inaktivität und Mitgefühlszufriedenheit bei Psycholog:innen in Weiterbildung sowie Burn-out-Symptomen und körperlicher Gesamtaktivität bei Psychologischen Psychotherapeut:innen waren keine Zusammenhänge zwischen körperlicher Aktivität und berufsbezogener Lebensqualität ersichtlich.

**Schlussfolgerungen:**

Psycholog:innen scheinen zu großen Teilen eine körperlich aktive und tendenziell zufriedene Berufsgruppe darzustellen. Zusammenhänge zwischen körperlicher Aktivität und Lebensqualität könnten teilweise durch Deckeneffekte verdeckt sein. Des Weiteren könnte eine Analyse der Ursachen für diese adaptiven Ausprägungen in Aktivität und Lebensqualität Anhaltspunkte für Maßnahmen zur Förderung anderer Professionen liefern.

## Abstract (en)

**Background:**

Clinical psychologists are often exposed to numerous stressors in their work with people with mental disorders, which can resonate in psychological stress and a reduced quality of life. Physical activity could be a protective resource but there is a lack of empirical evidence in this respect.

**Methods:**

In a cross-sectional online survey via SoSci-Survey (January–April 2020), physical activity as well as occupation-related quality of life (positive dimension: compassion satisfaction, negative dimension: compassion fatigue) were assessed in a convenience sample of 443 clinical psychologists using established self-report questionnaires (international physical activity questionnaire-short form, IPAQ-SF; professional quality of life scale, ProQOL).

**Results:**

The majority of the psychologists surveyed met the minimum level of physical activity recommended by the World Health Organization. On average, compassion satisfaction was relatively high, whereas relatively low to moderate levels of burnout symptoms and secondary traumatic stress (facets of compassion fatigue) were reported. Except for scattered small negative correlations between physical inactivity and compassion satisfaction among psychotherapists in training as well as burnout symptoms and overall physical activity among psychological psychotherapists, no associations between physical activity and profession-related quality of life were evident.

**Conclusion:**

Psychologists mostly seem to represent a physically active profession with a tendentially high occupation-related quality of life. In this context, associations between physical activity and quality of life could be partly hidden by ceiling effects. In addition, an analysis of the reasons for these adaptive characteristics in activity and professional quality of life could provide clues for supportive measures for other professions.

Obwohl bereits vor Jahrzehnten Studien zu berufsbezogenen Belastungen bei Psycholog:innen publiziert wurden, handelt es sich um ein in Forschung und Gesellschaft eher vernachlässigtes Thema. Psycholog:innen sind in ihrer Arbeit oft zahlreichen Stressoren ausgesetzt; diese scheinen nicht selten in psychische Belastung und eine verringerte Lebensqualität zu münden. Körperliche Aktivität könnte in diesem Kontext eine leicht verfügbare, puffernde Ressource darstellen; hierzu existieren jedoch bisher kaum Befunde.

## Hintergrund und Fragestellung

Die Arbeit mit Menschen mit psychischen Erkrankungen bedeutet die wiederholte Auseinandersetzung mit Belastungen, Erlebnissen und interaktiven Herausforderungen. Dies sowie strukturelle und wirtschaftliche Stressoren (hohe Arbeitsbelastung etc.) scheinen bei klinisch tätigen Psycholog:innen und anderen Gesundheitsfachkräften teilweise mit psychischen Belastungen sowie erheblichen Beeinträchtigungen der berufsbezogenen und allgemeinen Lebensqualität assoziiert zu sein (Allwood et al. 2022; Reimer et al. 2005; Stamm 2010; Thoreson et al. 1989). Beispielsweise berichteten über die Hälfte (56 %) der 460 befragten Sozialarbeiter:innen und Psycholog:innen in der US-amerikanischen Studie von Acker (2012) moderate bis hohe emotionale Erschöpfung (eine Facette von Burn-out). Besonders belastet waren im Mittel diejenigen, die mit stark traumatisierten und beeinträchtigten Individuen (Acker 2012) sowie Menschen mit Persönlichkeitsstörungen (Garcia et al. 2016) arbeiteten.

Während einzelne soziodemografische (z. B. weibliches Geschlecht, jüngeres Lebensalter; Allwood et al. 2022; McCormack et al. 2018) sowie persönlichkeitsbezogene Risikofaktoren (z. B. ausgeprägter Perfektionismus; D’Souza et al. 2011; Simpson et al. 2019) im Zusammenhang mit berufsbezogenen Belastungen bei Psycholog:innen und anderen Gesundheitsfachkräften analysiert wurden, sind Untersuchungen zu protektiven Variablen – insbesondere auf Verhaltensebene – noch spärlich gesät. Eine protektive stresspuffernde Verhaltensweise könnte beispielsweise in körperlicher Aktivität bestehen: In einer Studie zu Beginn der COVID-19-Pandemie in New York stellte körperliche Aktivität beispielsweise die am häufigsten (von 59 % der Gesundheitsfachkräfte) angegebene Coping-Strategie dar, um berufs- und pandemiebedingte Belastungen abzumildern (Shechter et al. 2020). Umgekehrt war geringere Aktivität zudem prospektiv mit mehr Stress sowie Angst- und depressiven Symptomen assoziiert (Jonsdottir et al. 2010). Aktuelle gezielte Untersuchungen zu den Ausprägungen ausgeübter körperlicher Aktivität und ihren Zusammenhängen zu berufsbezogener Lebensqualität sind bisher allerdings nicht vorhanden.

Metaanalysen über korrelative und quasiexperimentelle Studien aus der Allgemeinbevölkerung legen jedoch nahe, dass regelmäßige körperliche Aktivität mit höherem psychischem Wohlbefinden und besserer Lebensqualität zusammenhängt (Buecker et al. 2021; Chekroud et al. 2018). Auch bezüglich der positiven Auswirkungen von körperlicher Aktivität auf die Reduktion psychopathologischer Symptome bei Menschen mit verschiedenen psychischen Erkrankungen existiert umfangreiche Evidenz (Bendau et al. 2022a, b; Petzold et al. 2020; Ströhle et al. 2022; Stubbs und Rosenbaum 2018). Dagegen sind körperliche Aktivität und ihr potenzieller Nutzen bei denjenigen, die maßgeblich in die psychotherapeutische Arbeit mit Menschen mit psychischen Erkrankungen involviert sind, noch ein weitestgehend unerforschtes Thema.

Aus dieser Forschungslücke ergab sich die Zielstellung der vorliegenden Arbeit, das körperliche Aktivitätsniveau von Psycholog:innen in Deutschland zu untersuchen und Zusammenhänge mit berufsbezogener Lebensqualität (Mitgefühlszufriedenheit, -müdigkeit) zu analysieren. Es wurden positive Korrelationen zwischen körperlicher Aktivität und der als adaptiv bewerteten Dimension Mitgefühlszufriedenheit sowie negative Korrelationen mit den aversiv eingestuften Facetten der Mitgefühlsmüdigkeit, Burn-out-Symptomatik und sekundärer traumatischer Stress, erwartet.

## Methoden

### Studiendesign

Die Daten zu soziodemografischen Informationen, körperlicher Aktivität und berufsbezogener Lebensqualität wurden im Rahmen von 2 Teilprojekten einer querschnittlichen Online-Befragung über die Plattform SoSci-Survey erhoben. Die Studie wurde im Vorfeld von der Ethikkommission der Charité – Universitätsmedizin Berlin (EA01/034/19) genehmigt und auf clinicaltrials.gov registriert (NCT04165733, NCT04149912).

Die nonprobabilistische Rekrutierung der Proband:innen erfolgte deutschlandweit über Mailing-Listen von psychiatrischen Kliniken, psychotherapeutischen Ambulanzen und Ausbildungsinstituten sowie über die Verbreitung von Informationsmaterial in Klinikbesprechungen. Ein Berufsabschluss als Psycholog:in sowie eine aktuelle Tätigkeit in der Arbeit mit Menschen mit psychischen Erkrankungen stellten neben Volljährigkeit und ausreichenden Deutschkenntnissen zur Fragebogenbeantwortung die Einschlusskriterien dar. Die Teilnahme wurde nicht vergütet. Alle Proband:innen erklärten vor der Studienteilnahme ihr informiertes Einverständnis, und die Datenerhebung erfolgte vollständig anonym. Die Befragung fand von Januar bis April 2020 statt; es klickten 714 Personen auf den Fragebogenlink, und eine Gesamtzahl von 443 Psycholog:innen, die alle relevanten Informationen ausgefüllt hatten, wurde in den finalen Analysen berücksichtigt.

### Messinstrumente

#### Kurzfassung des International Physical Activity Questionnaire

Um das Ausmaß körperlicher Aktivität standardisiert zu erfassen, wurde die Kurzfassung des International Physical Activity Questionnaire (IPAQ-SF) angewendet (IPAQ Research Committee 2005). Der IPAQ-SF erfasst 3 definierte Aktivitätsformen (intensiv-anstrengende körperliche Aktivität, moderat-anstrengende Aktivität, Zufußgehen) sowie die durchschnittliche Zeit im Sitzen pro Tag und pro Woche. Als Gesamt-Score wurde die Gesamtminutenzahl körperlicher Aktivität (Summe der 3 Aktivitätsformen) und für eine gewichtete Quantität die Gesamtzahl der MET-Minuten (Aktivitätsformen jeweils mit ihrem metabolischen Äquivalent [MET]^1^ multipliziert und addiert) herangezogen. Neben diesen kontinuierlichen Parametern erfolgte zudem anhand der MET-Minuten gemäß der IPAQ-SF-Auswertungsleitlinie (IPAQ Research Committee 2005) eine Kategorisierung in inaktive (< 600 MET-min/Woche), minimal-aktive (600-2999 MET-min/Woche) und aktive Teilnehmende (≥ 3000 MET-min/Woche).

#### Professional Quality of Life Scale

Bezüglich beruflicher Lebensqualität wurde die 30 Items umfassende Professional Quality of Life Scale eingesetzt (ProQOL; Stamm 2010; die deutsche Übersetzung von Gräßer, Hovermann und Kebé erfolgte 2016, eine detaillierte psychometrische Überprüfung der deutschsprachigen Version steht noch aus). Diese erfasst *Mitgefühlszufriedenheit* (Subskala: Compassion Satisfaction Scale, CS) und *Mitgefühlsmüdigkeit*, die sich wiederum aus den Subkomponenten *Burn-out* (Burn-out Scale, BO) und *sekundärer traumatischer Stress* (Secondary Traumatic Stress Scale, STS) zusammensetzt (Stamm 2010). Jede der 3 Skalen umfasst 10 Items, die auf einer 5‑stufigen Likert-Skala von 1 („nie“) bis 5 („sehr oft/immer“) bewertet werden. Die Subskalen-Summen-Scores erstrecken sich von 10–50, wobei die Level der Mitgefühlszufriedenheit bzw. der Burn-out-Symptomatik und des sekundären traumatischen Stresses in niedrig (10–22), moderat (23–41) und hoch (41–50) klassifiziert werden (Stamm 2010).

Die Reliabilität der 3 Subskalen fiel in der vorliegenden Stichprobe akzeptabel bis gut aus, wenngleich etwas niedriger als in der englischsprachigen Originalversion (CS: interne Konsistenz: α = 0,87 vs. 0,88; BO: α = 0,73 vs. 0,75; STS: α = 0,69 vs. 0,81; Stamm 2010).

### Analysen

Alle quantitativen Analysen erfolgten mithilfe des Statistikprogramms IBM SPSS Statistics Version 29. Neben deskriptiven Analysen wurden inferenzstatistisch querschnittliche Zusammenhänge der körperlichen Betätigung mit beruflicher Lebensqualität mithilfe nonparametrischer Partialkorrelationen (Herauspartialisierung von Alter und Geschlecht) berechnet. Dieses Vorgehen wurde aufgrund verletzter Normalverteilungsannahmen sowie angesichts signifikanter Zusammenhänge von Alter und Geschlecht mit körperlicher Aktivität sowie berufsbezogener Belastung in vorherigen Studien gewählt. Fehlende Werte wurden listenweise ausgeschlossen und das Signifikanzniveau (2-seitig) auf 0,05 festgesetzt.

## Ergebnisse

### Stichprobe

Von den insgesamt 443 Teilnehmenden identifizierten sich 87,1 % (*n* = 386) als weiblich und 12,9 % (*n* = 57) als männlich; das durchschnittliche Lebensalter betrug 36,81 Jahre (SD ± 10,73 Jahre; Range 24 bis 74 Jahre). Die Stichprobe setzte sich aus klinisch tätigen Psycholog:innen ohne Psychotherapieweiterbildung (4,5 %; *n* = 20); klinisch tätigen Psycholog:innen in Therapieweiterbildung (57,8 %; *n* = 256) und Psychologischen Psychotherapeut:innen (37,7 %; *n* = 167) zusammen. Bezüglich des Arbeitssettings berichteten 59,6 % (*n* = 264) der Teilnehmenden, vorwiegend ambulant, und 40,4 % (*n* = 179) der Teilnehmenden, vorwiegend stationär tätig zu sein. Als Arbeitskontext wurden am häufigsten psychiatrische Kliniken (24,4 %) und ambulante Behandlungen im Weiterbildungsrahmen (25,3 %) genannt (psychiatrische Universitätsklinik: 8,4 %, andere Klinik: 16,5 %; niedergelassen/ambulant selbstständig mit eigenem Kassensitz: 15,1 %, in Anstellung: 6,3 %, ohne eigenen Kassensitz, privat bzw. Kostenerstattung: 4,1 %). Die Berufserfahrung erstreckte sich von einem Jahr bis zu 44 Jahren mit einem Mittelwert von 8,79 Jahren (SD ± 8,49 Jahre).

### Körperliche Aktivität

Indikatoren für die in der letzten Woche absolvierte körperliche Aktivität laut den Items des IPAQ-SF sind in Tab. 1 aufgeführt. Laut den selbstberichteten Angaben verweilten die Teilnehmenden im Durchschnitt etwa 8 h/Tag im Sitzen. Etwa 5 h/Woche wurden im Schnitt mit Zufußgehen verbracht, 3 h mit moderater körperlicher Aktivität und 2,5 h mit intensiv-anstrengender körperlicher Aktivität. Einzelne besonders aktive Individuen scheinen die Mittelwerte zu erhöhen; die Medianwerte waren mit 3 h Zufußgehen und jeweils 2 h moderater und intensiver Aktivität niedriger.

**Table 1:** 

Kennwert	Sitzen (min/Tag)	Zufußgehen (min/Woche)	Moderate Aktivität (min/Woche)	Intensive Aktivität (min/Woche)	Gesamtaktivität (min/Woche)	Zufußgehen (MET-min/Woche)	Moderate Aktivität (MET-min/Woche)	Intensive Aktivität (MET-min/Woche)	Gesamtaktivität (MET-min/Woche)
*Gesamtstichprobe (n* *=* *443)*									
M (± SD)	477 (± 174)	295 (± 334)	183 (± 255)	151 (± 175)	630 (± 509)	974 (± 1105)	735 (± 1022)	1211 (± 1400)	2921 (± 2337)
Range	120–1440	0–2520	0–2520	0–1680	5–3360	0–8316	0–10.080	0–13.440	20–18.186
10. Perzentil	300	40	0	0	180	132	0	0	798
25. Perzentil	360	90	41	40	311	297	165	320	1398
Median	480	180	120	120	480	594	480	960	2394
75. Perzentil	600	420	240	180	836	1386	960	1440	3795
90. Perzentil	660	720	420	360	1200	2376	1680	2880	5647
*Klinisch tätige Psycholog:innen in Psychotherapieweiterbildung (n* *=* *256)*									
M (± SD)	481 (± 166)	310 (± 361)	167 (± 248)	158 (± 196)	636 (± 540)	1024 (± 1192)	669 (± 993)	1267 (± 1565)	2960 (± 2528)
Median	480	205	100	120	480	677	400	960	2361
*Psychologische Psychotherapeut:innen (n* *=* *167)*									
M (± SD)	475 (± 189)	263 (± 283)	202 (± 268)	146 (± 147)	612 (± 462)	869 (± 934)	810 (± 1073)	1165 (± 1174)	2844 (± 2065)
Median	420	150	150	95	510	495	600	760	2465
*Vorwiegend ambulant tätig (n* *=* *179)*									
M (± SD)	475 (± 178)	260 (± 294)	192 (± 266)	145 (± 147)	597 (± 455)	857 (± 969)	768 (± 1065)	1162 (± 1176)	2787 (± 1994)
Median	480	150	120	100	485	495	480	800	2396
*Vorwiegend stationär tätig (n* *=* *264)*									
M (± SD)	482 (± 171)	348 (± 383)	172 (± 240)	161 (± 210)	681 (± 578)	1150 (± 1264)	688 (± 959)	1286 (± 1681)	3123 (± 2764)
Median	480	210	90	120	480	693	360	960	2340

Anstrengende Aktivitäten bezeichnen laut der Kurzfassung des International Physical Activity Questionnaire (IPAQ-SF) Aktivitäten, die starke körperliche Anstrengungen erfordern (z. B. schweres Heben, Graben, Aerobic, schnelles Fahrradfahren). Moderate Aktivitäten umfassen weniger anstrengende Aktivitäten, bei denen die Atmung leicht beschleunigt ist (z. B. Tragen leichter Lasten, Fahrradfahren bei gewöhnlicher Geschwindigkeit)

Die Gruppe der klinisch tätigen Psycholog:innen ohne Weiterbildung wurde aufgrund der kleinen Gruppengröße nicht dargestellt

*M* Mittelwert, *MET* „metabolic equivalents of task“, *SD* Standardabweichung

Laut dem angegebenen Energieumsatz (MET-min/Woche) wurde nur ein kleiner Prozentsatz (6,8 %; *n* = 30) der Stichprobe als *inaktiv* klassifiziert (und wies entsprechend ein Bewegungsausmaß unterhalb der Mindestempfehlungen der Weltgesundheitsorganisation auf; Ding et al. 2020), während 56,2 % (*n* = 249) als *minimal aktiv* und 36,6 % (*n* = 162) als* aktiv* eingestuft wurden. Weder im Hinblick auf diese Kategorien noch auf Mittel- und Medianwerte (Tab. 1) zeigten sich substanzielle Unterschiede zwischen Psycholog:innen in Psychotherapieweiterbildung vs. Psychologischen Psychotherapeut:innen sowie zwischen vorwiegend ambulant vs. vorwiegend stationär tätigen Psycholog:innen.

### Körperliche Aktivität und berufsbezogene Lebensqualität

Die Mitgefühlszufriedenheit in der Stichprobe fiel mit einem Mittelwert von 39,16 (SD ± 4,90; Range 16–49) relativ hoch aus; nur 2 Personen wurden in die niedrig zufriedene Kategorie eingestuft, zwei Drittel (65,7 %) waren mittelmäßig zufrieden und ein Drittel (33,7 %) war hoch zufrieden.

Der Burn-out-Score betrug im Durchschnitt 20,60 (SD ± 4,14; Range: 12–36) und sekundärer traumatischer Stress im Mittel 17,25 (SD ± 3,57; Range: 10–30). Bezüglich dieser beiden Subskalen der Komponente Mitgefühlsmüdigkeit berichtete die Mehrheit der Teilnehmenden niedrige Ausprägungen (BO: niedrig: 74,5 %, moderat: 25,5 %; STS: niedrig: 91,4 %, moderat: 8,6 %), und keine Person überschritt den Schwellenwert zu als hoch klassifizierter Belastung.

In der Gesamtstichprobe waren keine linearen Zusammenhänge zwischen Parametern körperlicher Aktivität und den Skalen des Fragebogens zu berufsbezogener Lebensqualität ersichtlich (Tab. 2). Bei der differenzierten Betrachtung der Subgruppe der Psycholog:innen in Psychotherapieweiterbildung zeigte sich dagegen hypothesenkonform ein signifikant negativer Zusammenhang der täglich im Sitzen verbrachten Zeit mit Mitgefühlszufriedenheit. In der Subgruppe der Psychologischen Psychotherapeut:innen waren des Weiteren erwartungskonform negative Zusammenhänge zwischen der Gesamtaktivität in Minuten sowie MET-min/Woche mit Burn-out-Symptomen beobachtbar. Die Effektgröße der Zusammenhänge ist als klein zu bewerten, und zudem gilt es, die α‑Fehler-Kumulation durch multiples Testen zu berücksichtigen.

**Table 2:** 

	Sitzen (min/Tag)	Gesamtaktivität (min/Woche)	Zufußgehen (MET-min/Woche)	Moderate Aktivität (MET-min/Woche)	Intensive Aktivität (MET-min/Woche)	Gesamtaktivität (MET-min/Woche)
*Gesamtstichprobe (n* *=* *443)*						
Mitgefühlszufriedenheit	−0,040 (0,410)	0,073 (0,131)	0.070 (0,147)	0,015 (0,752)	0,074 (0,124)	0,077 (0,111)
Burn-out	0,046 (0,342)	−0,033 (0,495)	0,012 (0,808)	−0,001 (0,982)	−0,064 (0,185)	−0,042 (0,380)
Sekundärer traumatischer Stress	0,009 (0,848)	0,066 (0,170)	0,063 (0,188)	0,036 (0,456)	0,023 (0,635)	0,064 (0,183)
*Klinisch tätige Psycholog:innen in Psychotherapieweiterbildung (n* *=* *256)*						
Mitgefühlszufriedenheit	−0,156 (0,013)*	0,071 (0,260)	0,068 (0,285)	0,018 (0,776)	0,106 (0,095)	0,082 (0,195)
Burn-out	0,079 (0,213)	0,066 (0,295)	0,066 (0,296)	0,033 (0,599)	−0,042 (0,512)	0,047 (0,461)
Sekundärer traumatischer Stress	−0,033 (0,599)	0,102 (0,107)	0,053 (0,405)	0,043 (0,494)	0,086 (0,173)	0,110 (0,081)
*Psychologische Psychotherapeut:innen (n* *=* *167)*						
Mitgefühlszufriedenheit	0,103 (0,190)	0,109 (0,166)	0,110 (0,163)	0,015 (0,851)	0,047 (0,557)	0,103 (0,194)
Burn-out	0,043 (0,590)	−0,161 (0,041)*	−0,075 (0,340)	−0,079 (0,318)	−0,042 (0,512)	−0,154 (0,050)*
Sekundärer traumatischer Stress	0,077 (0,329)	0,055 (0,486)	−0,057 (0,468)	0,028 (0,720)	0,086 (0,173)	0,036 (0,647)

*p* in Klammern, *p < 0,05; signifikante Werte sind fettgedruckt. In der Gruppe der klinisch tätigen Psycholog:innen ohne Weiterbildung wurden aufgrund der kleinen Gruppengröße keine separaten Zusammenhänge berechnet

## Diskussion

### Zusammenfassung und Interpretation der Befunde

Insgesamt fiel das Ausmaß der körperlichen Aktivität in der betrachteten Psycholog:innen-Stichprobe moderat hoch aus; nur wenige Teilnehmende erzielten Werte unter den Empfehlungen der Weltgesundheitsorganisation bezüglich eines Mindestmaßes an körperlicher Aktivität pro Woche (mindestens 600 MET-min/Woche; Ding et al. 2020). Eine Gesamtzahl von etwa 630 min Aktivität/Woche bzw. im Schnitt 90 min täglich fiel vergleichbar mit Befunden repräsentativer Allgemeinbevölkerungsstichproben aus Deutschland, Frankreich, Kanada und den USA aus. Dort betrug die durchschnittliche Bewegung pro Tag 80–105 min (Graf und Cecchini 2019), während andere Untersuchungen teils deutlich niedrigere Bewegungslevel ergaben (Ding et al. 2020; Stubbs und Rosenbaum 2018).

Die Indikatoren für berufsbezogene Lebensqualität waren im Schnitt positiv ausgeprägt – die Mitgefühlszufriedenheit war tendenziell hoch, während eher niedrige bis moderate Anzeichen von Burn-out sowie sekundärem traumatischem Stress berichtet wurden. Verglichen mit den gemittelten Skalen-Scores einer Übersichtsarbeit (La de Rosa et al. 2018) über 30 Studien mit Gesundheitsfachkräften verschiedener Professionen (Pflegepersonal, ärztliches Personal, Sozialarbeiter:innen, Musiktherapeut:innen etc.) fiel die Mitgefühlszufriedenheit in der vorliegenden psychologischen Stichprobe im Durchschnitt geringfügig höher aus (M = 39,2 vs. M = 37,7; Range der Einzelstudien: M = 29,2–42,4), während Burn-out-Symptome (20,6 vs. 22,8; Range der Einzelstudien: 15,2–32,3) und sekundärer traumatischer Stress (16,7 vs. 17,3; Range der Einzelstudien: 5,8–27,0) minimal niedriger ausgeprägt waren. Es handelt sich jedoch um rein deskriptive Vergleiche; zukünftige Studien könnten hier ansetzen und die berufsbezogene Lebensqualität systematisch inferenzstatistisch zwischen verschiedenen Berufsgruppen und Arbeitskontexten vergleichen. Des Weiteren gilt es, verschiedene soziodemografische (z. B. Alter, Geschlechterverteilung) und tätigkeitsbezogene Faktoren zu berücksichtigen. So könnte die eher hohe Zufriedenheit in der vorliegenden Stichprobe u. a. auf den großen Anteil ambulant tätiger Psycholog:innen (häufig weniger belastend empfunden als stationäre Settings; Allwood et al. 2022) sowie Psycholog:innen in Psychotherapieweiterbildung (die sich noch am Anfang ihres psychotherapeutischen Berufslebens befinden und möglicherweise besonders motiviert sind) zurückführbar sein.

Die in den Subgruppenanalysen vereinzelt beobachteten negativen Zusammenhänge zwischen Inaktivität und Mitgefühlszufriedenheit sowie körperlicher Aktivität und Burn-out-Symptomatik geben einen ersten Hinweis darauf, dass körperliche Aktivität hypothesenkonform eine adaptive Funktion für die berufsbezogene Lebensqualität einnehmen könnte. Dem sollte in weiterführenden (bestenfalls experimentellen) Untersuchungen vertiefend nachgegangen werden, und, falls sich diese Befunde bestätigen lassen, sollten praktische Handlungsempfehlungen abgeleitet werden.

Gleichzeitig gilt es aber auch zu diskutieren, weshalb die vorgestellten Daten insgesamt die erwarteten Zusammenhänge zwischen körperlicher Aktivität und berufsbezogener Lebensqualität überwiegend nicht bestätigen konnten. Möglicherweise kann das Ausbleiben signifikanter linearer Zusammenhänge durch inhaltliche und methodische Gegebenheiten erklärt werden. Beispielsweise könnten Bereichsbeschränkungen und Deckeneffekte durch die eher moderat bis hoch ausgeprägten Parameter körperlicher Aktivität und berufsbezogener Lebensqualität das Erkennen bestehender Zusammenhänge verhindern. Eventuell sind die Zusammenhänge überdies nicht linear, sondern beispielsweise umgekehrt U‑förmig (falls sowohl zu wenig als auch exzessiv viel Bewegung mit geringerer Lebensqualität assoziiert ist) oder anders gestaltet. Des Weiteren ist es ggf. ungünstig, dass der Fragebogen zur Erfassung körperlicher Aktivität eine andere Zeitspanne betrifft als das Instrument bezüglich berufsbezogener Lebensqualität (7 vs. 30 Tage). Gerade in Bezug auf die körperliche Aktivität ist zudem das regelmäßige Bewegungsausmaß über einen längeren Zeitraum hinweg möglicherweise noch aussagekräftiger als nur ein kleines einwöchiges Zeitfenster. Außerdem weisen beide Instrumente gewisse methodische Kritikpunkte auf: Das Antwortformat des IPAQ-SF ist für versehentliche fehlerhafte Angaben anfällig, die Zeiten im Sitzen werden häufig unterschätzt (IPAQ Research Committee 2005), und bezüglich der beiden Skalen zur Mitgefühlsmüdigkeit des ProQOL werden reliabilitäts- und validitätsbezogene methodische Schwächen bemängelt (Heritage et al. 2018). Es handelt sich überdies um Selbstberichtfragebogen, die für bewusste sowie unbewusste Erinnerungs‑, Wahrnehmungs- und Antwortverzerrungen (z. B. soziale Erwünschtheit) empfänglich sind. Diesbezüglich wäre in zukünftigen Untersuchungen eine Ergänzung mit anderen Messinstrumenten – beispielsweise gekoppelt mit objektiven Messmöglichkeiten, wie z. B. Pedometerdaten – wünschenswert.

Zudem sind evtl. andere Aspekte berufsbezogener und allgemeiner Lebensqualität stärker mit körperlicher Aktivität assoziiert als Mitgefühlszufriedenheit und -müdigkeit. Umgekehrt könnten sich andere Aspekte des privaten und beruflichen Lebens stärker in der (berufsbezogenen) Lebensqualität niederschlagen, sodass der Effekt körperlicher Aktivität im Vergleich nicht mehr sichtbar wird (Simpson et al. 2019). Auch diesbezüglich könnten zukünftige Studien bestenfalls weitere Variablen integrieren. Des Weiteren ist möglicherweise eine genauere Differenzierung verschiedener Formen körperlicher Aktivität zielführend – beispielsweise in Freizeitaktivitäten vs. Aktivitäten im Berufs- und im Haushaltskontext. Letztere wiesen in einer Untersuchung von McKercher et al. (2009) im Mittel weniger gute Assoziationen mit psychischer Gesundheit auf als Erstere.

Psycholog:innen mit auffallend niedrigen Aktivitätsausprägungen sollte ggf. besondere Aufmerksamkeit zuteilwerden, da körperliche Inaktivität das Risiko für körperliche und psychische Beschwerden deutlich erhöht (Bendau et al. 2022b; Stubbs und Rosenbaum 2018). Ebenso ist für Individuen mit besonders niedriger berufsbezogener Lebensqualität die Schaffung adäquater Unterstützungsmöglichkeiten ratsam (Graf und Cecchini 2019). Gleichzeitig ist es bemerkenswert, dass die Stichprobe insgesamt relativ aktiv und beruflich zufrieden ausfiel. Sollte sich die Evidenz dafür in weiteren Untersuchungen erhärten, könnte dies einen Ausgangspunkt dafür liefern zu eruieren, welche Faktoren zu Aktivität, Resilienz und Zufriedenheit dieser Berufsgruppe beitragen, und daraus könnten möglicherweise Erkenntnisse für die Gesundheitsförderung anderer Professionen abgeleitet werden.

### Limitationen der Studie

Es gilt zu beachten, dass es sich bei der Gruppe der Teilnehmenden der vorliegenden Studie um eine Gelegenheitsstichprobe handelt, deren Repräsentativität für die Grundgesamtheit der Psycholog:innen eingeschränkt sein könnte. Beispielsweise ist zu berücksichtigen, dass die Mehrheit der Stichprobe weiblich, eher jung, noch in Psychotherapieweiterbildung und ambulant tätig war und somit ggf. die Generalisierbarkeit auf Individuen mit anderen soziodemografischen und tätigkeitsbezogenen Merkmalen verringert ist. Personen mit Interesse am Studienthema sowie Online-Medien-affine Individuen wiesen z. B. ggf. eine höhere Teilnahmewahrscheinlichkeit auf. Außerdem sind durch das querschnittliche korrelativ-observationale Studiendesign keinerlei kausale Schlussfolgerungen möglich, und verzerrende Einflüsse von Drittvariablen können nicht ausgeschlossen werden. Entsprechend sind für die Zukunft insbesondere randomisierte, kontrollierte und längsschnittliche Studien wünschenswert.

### Resümee

Die vorliegende Studie stellt durch die erstmalige Untersuchung von körperlicher Aktivität und berufsbezogener Lebensqualität bei Psycholog:innen in Deutschland mithilfe etablierter Instrumente einen wichtigen Fortschritt bezüglich dieser relevanten und bisher noch unterbeforschten Thematik dar und legt einen Grundstein für Nachfolgeuntersuchungen.

## Fazit für die Praxis

- Der Großteil der befragten klinisch tätigen Psycholog:innen erfüllte weitestgehend das von der Weltgesundheitsorganisation empfohlene Mindestmaß an körperlicher Aktivität.
- Mitgefühlszufriedenheit als positive Dimension berufsbezogener Lebensqualität war bei den klinisch tätigen Psycholog:innen im Durchschnitt relativ hoch ausgeprägt.
- Im Mittel wurden eher geringe Ausprägungen von Burn-out-Symptomatik sowie sekundärem traumatischem Stress (Facetten der aversiven Lebensqualitätkomponente Mitgefühlsmüdigkeit) berichtet.
- Außer vereinzelten negativen Korrelationen zwischen Inaktivität und Mitgefühlszufriedenheit bei Psycholog:innen in Weiterbildung sowie Burn-out-Symptomen und körperlicher Gesamtaktivität bei Psychologischen Psychotherapeut:innen waren keine Zusammenhänge zwischen körperlicher Aktivität und berufsbezogener Lebensqualität ersichtlich. Dies kann in verschiedenen methodischen und inhaltlichen Aspekten begründet sein.
- Zukünftige Analysen und Studien sollten die potenzielle protektive Rolle von körperlicher Aktivität bezüglich berufsbedingter Stressoren und Belastungen weitergehend untersuchen.

## Footnotes

1 Das metabolische Äquivalent (MET) beschreibt das Verhältnis des Energieumsatzes während körperlicher Aktivität zum Ruheenergieumsatz. Das MET von moderater Aktivität (z. B. langsames Schwimmen, Fahrradfahren) beträgt z. B. 4 – inhaltlich bedeutet dies, dass bei moderater Aktivität 4‑mal so viel Energie umgesetzt wird wie im Ruhezustand. Das MET lässt sich unterschiedlich berechnen, z. B. über die Sauerstoffaufnahme oder den Energieverbrauch (Kilokalorien). In der vorliegenden Studie wurden die IPAQ-Normwerte verwendet (Zufußgehen: 3,3; moderate Aktivität: 4; intensive Aktivität: 8; IPAQ Research Committee 2005). Eine halbe Stunde Zufußgehen entspricht also 99 (30 min • 3,3) MET-min; eine halbe Stunde langsames Schwimmen 120 (30 min • 4) MET-min.
